# Combined Effects of Having Sleep Problems and Taking Sleeping Pills on the Skeletal Muscle Mass and Performance of Community-Dwelling Elders

**DOI:** 10.1038/s41598-019-50295-w

**Published:** 2019-09-24

**Authors:** Chuan-Wei Yang, Chia-Ing Li, Tsai-Chung Li, Chiu-Shong Liu, Chih-Hsueh Lin, Wen-Yuan Lin, Cheng-Chieh Lin

**Affiliations:** 1 Department of Medical Research, China Medical University Hospital, Taichung, Taiwan; 2 School of Medicine, College of Medicine, China Medical University, Taichung, Taiwan; 3 Department of Public Health, College of Public Health, China Medical University, Taichung, Taiwan; 4 Department of Healthcare Administration, College of Medical and Health Sciences, Asia University, Taichung, Taiwan; 5 Department of Family Medicine, China Medical University Hospital, Taichung, Taiwan

**Keywords:** Sleep disorders, Geriatrics

## Abstract

This study aimed to explore the combined effects of having sleep problems and taking sleeping pills on the skeletal muscle mass and performance of community-dwelling elders. A total of 826 participants who have complete information regarding dual-energy X-ray absorptiometry examination, questionnaire, and physical performance tests were included. The status of having sleep problems and taking sleeping pills was assessed with a self-reported questionnaire. The prevalence rates of sleep problems among older men and women were 37.4% and 54.5%, respectively. After multivariate adjustment, the mean height-adjusted skeletal muscle indices for elders having sleep problems and taking sleeping pills among men and women were 7.29 and 5.66 kg/m^2^, respectively, which were lower than those without sleep problems (*P* = 0.0021 and *P* = 0.0175). The performance of the older men having sleep problems and taking sleeping pills in terms of walking speed, grip strength, and number of squats, was poorer than those of the older men without sleep problems. The status of having sleep problems and taking sleeping pills was correlated with low skeletal muscle mass and poor physical performance in community-dwelling elders. These findings suggest that having sleep problems and taking sleeping pills are associated with having sarcopenia among community elderly.

## Introduction

Sleep problems, such as experiencing difficulty in falling asleep, having many dreams, becoming easily awakened, and suffering from sleepiness, are common among elderly. The prevalence of insomnia among community-dwelling elderly in Taiwan is 41%^1^, and it is higher in females (63.3%) than in males (36.7%). In the US, 42% of 9000 elders experience difficulty in falling asleep or maintaining sleep^2^. In Hong Kong, the prevalence of sleep disturbance is quite high (42.2%)^3^.

According to Taiwan Food and Drug Administration (TFDA) statistics, the Taiwanese consumed 339 million sleeping pills in 2014, and the highest proportion of individuals taking sleeping pills constitutes older females. Taking sleeping pills may elicit side effects, including dizziness, daytime drowsiness, diarrhea, constipation, and difficulty in keeping balance. Furthermore, a 10-year follow-up study found that the high cumulative use of anticholinergic drugs is associated with the increased risk of Alzheimer’s disease and dementia among the elderly^4^. Therefore, sleep problems among the elderly are crucial health issues.

Sleep problems in the elderly contribute to numerous health effects and increase the risk of adverse outcomes, such as falls^5,6^, poor quality of life^7^, nursing home placement^8^, depression^9^, cognitive decline^10^, and even death^8^. A previous study showed that the lower the score in each domain of Short Form Health Survey (SF-36) is the greater the number of reported insomnia among the elderly^7^. In Singapore, elders with sleep problems are associated with a significantly high risk of depression^9^. Moreover, subjects with sleep problems, especially waking up too early, experience a significant declined in cognitive functions compared with subjects without sleep problems during a 3-year follow-up^11^.

In addition to mental functions, the physical functions of the elderly are affected by sleep problems. In the US, elders with excessive daytime sleepiness have a significantly high risk of recurrent falls. Short sleep duration (≤5 hours) and poor sleep efficiency are related to the increased risk of falls^5^. After adjustments have been made for many potential variables, such as depression, cognitive function, and long- and short-acting benzodiazepine use, the association between sleep problems and risk of falls has been observed, but the potential mechanisms mediating such association among the elderly are unclear.

Muscle function among the elderly is an important factor related to falls^12^. A meta-analysis aggregated 16 studies to analyze the risk factors of falls and revealed that significant factors included muscle weakness, gait deficit, balance deficit, mobility limitation, visual deficit, and cognitive impairment^13^. Muscle weakness is another major risk factor of falls. However, whether muscle weakness as the potential mechanism of the association between having sleep problems and falls among the elderly is unclear. Previous study showed that sleep duration was correlated with testosterone levels among men^14^. Furthermore, some studies found that testosterone levels among older men was associated with muscle mass^15^ and physical performance^16^. But previous studies did not consider the combined effects of sleep problems and sleep-inducing drugs on skeletal muscle mass and performance. Therefore, we test the hypothesis that whether having sleep problems and taking sleeping pills are independently or jointly associated with skeletal muscle mass and physical performance indicators, such as walking speed and grip strength, among elderly.

## Results

### Characteristics of the study participants

The proportions of having sleep problems among elderly men and women were 37.4% and 54.5%, respectively. The major problem was difficulty in falling asleep. The sociodemographic factors, health-related practices, and chronic problems of the study subjects are shown in Table 1. The distributions of age, hypertension, diabetes mellitus, hyperlipidemia, and stroke among elderly men were significantly different among the groups with various sleep problems. The proportions of hypertension, diabetes mellitus, hyperlipidemia, and stroke in the group having sleep problems and taking sleeping pills were higher than those in the other groups of elderly men. Conversely, the distributions of alcohol drinking and exercise among elderly women were significantly different from the groups with various sleep problems. The proportions of alcohol drinking and exercise in the group having sleep problems and taking sleeping pills were lower than those of the group without sleep problems among elderly women. Moreover, the mean of GDS scores of the elderly men and women were significantly different among the groups with various sleep problems. In the elderly men and women, the mean GDS scores in the group having sleep problems and taking sleeping pills were higher than those in the two other groups.

**Table 1:** Characteristics of the Study Participants According to the Status of Having Sleep Problems and Taking Sleeping Pills.

	Sleep problems among men						*P* value	Sleep problems among women						*P* value
	No (n = 276)		Yes (n = 165)					No (n = 175)		Yes (n = 210)				
	n	(%)	Sleeping pills					n	(%)	Sleeping pills				
			No (n = 83)		Yes (n = 82)					No (n = 108)		Yes (n = 102)		
			n	(%)	n	(%)				n	(%)	n	(%)	
**Sociodemographic factors**														
Age							0.0257							0.0702
≦70	85	(30.8)	31	(37.4)	27	(32.9)		84	(48.0)	38	(35.2)	35	(34.3)	
70–75	59	(21.4)	28	(33.7)	16	(19.5)		48	(27.4)	31	(28.7)	29	(28.4)	
>75	132	(47.8)	24	(28.9)	39	(47.6)		43	(24.6)	39	(36.1)	38	(37.3)	
Education							0.1133							0.2181
Illiterate	11	(4.1)	3	(3.7)	7	(8.6)		28	(16.3)	15	(14.3)	23	(23.5)	
≦6 years	53	(19.6)	13	(16.1)	24	(29.6)		66	(38.6)	34	(32.4)	24	(24.5)	
7–12 years	98	(36.3)	32	(39.5)	20	(24.7)		54	(31.6)	40	(38.1)	33	(33.7)	
≧13 years	108	(40.0)	33	(40.7)	30	(37.0)		23	(13.5)	16	(15.2)	18	(18.4)	
**Health-related practices**														
Smoking							0.1239							0.4049
Never	180	(65.2)	55	(66.3)	42	(51.2)		173	(98.9)	103	(95.4)	99	(97.1)	
Current	36	(13.0)	14	(16.9)	15	(18.3)		1	(0.6)	2	(1.9)	2	(2.0)	
Former	60	(21.7)	14	(16.9)	25	(30.5)		1	(0.6)	3	(2.8)	1	(1.0)	
Drinking							0.3997							0.0014^a^
Never	186	(67.4)	54	(65.1)	55	(67.1)		161	(92.0)	104	(96.3)	100	(98.0)	
Current	62	(22.5)	19	(22.9)	13	(15.9)		14	(8.0)	4	(3.7)	—	—	
Former	28	(10.1)	10	(12.1)	14	(17.1)		—	—	—	—	2	(2.0)	
Exercise							0.7338							0.0356
No	60	(21.7)	20	(24.1)	21	(25.6)		37	(21.1)	37	(34.3)	31	(30.7)	
Yes	216	(78.3)	63	(75.9)	61	(74.4)		138	(78.9)	71	(65.7)	70	(69.3)	
**Chronic problem/Illness**														
Hypertension							0.0154							0.1862
No	135	(49.3)	48	(57.8)	28	(35.4)		89	(51.5)	59	(56.2)	44	(43.6)	
Yes	139	(50.7)	35	(42.2)	51	(64.6)		84	(48.5)	46	(43.8)	57	(56.4)	
Diabetes mellitus							0.0447							0.4675
No	222	(81.6)	73	(88.0)	59	(72.8)		148	(86.0)	90	(83.3)	91	(89.2)	
Yes	50	(18.4)	10	(12.0)	22	(27.2)		24	(14.0)	18	(16.7)	11	(10.8)	
Heart disease							0.1943							0.2925
No	194	(71.1)	62	(75.6)	51	(63.0)		125	(72.2)	74	(70.5)	62	(63.3)	
Yes	79	(28.9)	20	(24.4)	30	(37.0)		48	(27.8)	31	(29.5)	36	(36.7)	
Hyperlipidemia							0.0217							0.9201
No	220	(82.1)	72	(87.8)	57	(71.3)		114	(66.7)	74	(68.5)	66	(66.0)	
Yes	48	(17.9)	10	(12.2)	23	(28.7)		57	(33.3)	34	(31.5)	34	(34.0)	
Hyperuricemia							0.5364							0.6002
No	234	(85.7)	74	(90.2)	69	(85.2)		160	(93.0)	97	(91.5)	96	(95.1)	
Yes	39	(14.3)	8	(9.8)	12	(14.8)		12	(7.0)	9	(8.5)	5	(4.9)	
Arthritis							0.3618							0.2187
No	217	(81.0)	71	(87.6)	63	(80.8)		125	(74.4)	69	(67.7)	76	(78.4)	
Yes	51	(19.0)	10	(12.4)	15	(19.2)		43	(25.6)	33	(32.3)	21	(21.6)	
Stroke							0.0001							0.8114
No	254	(94.4)	80	(96.4)	64	(81.0)		166	(95.9)	99	(94.3)	95	(95.0)	
Yes	15	(5.6)	3	(3.6)	15	(19.0)		7	(4.1)	6	(5.7)	5	(5.0)	
Fall history							0.3690							0.0807
No	236	(85.8)	66	(79.5)	68	(82.9)		130	(74.3)	77	(72.0)	63	(61.8)	
Yes	39	(14.2)	17	(20.5)	14	(17.1)		45	(25.7)	30	(28.0)	39	(38.2)	
**Types of sleep problems**														
Difficulty falling asleep	—	—	41	(51.9)	63	(78.8)		—	—	66	(62.9)	85	(86.7)	
Many dreams	—	—	17	(21.5)	16	(20.0)		—	—	18	(17.1)	18	(18.4)	
Easy awakening	—	—	33	(41.8)	28	(35.0)		—	—	41	(39.4)	32	(32.7)	
Sleepiness	—	—	2	(2.5)	—	—		—	—	2	(1.9)	1	(1.0)	
Mental factors	Mean	(SD)	Mean	(SD)	Mean	(SD)		Mean	(SD)	Mean	(SD)	Mean	(SD)	
GDS-15 item	1.48	(1.81)	1.78	(1.91)	2.50	(2.80)	0.0004^b^	1.78	(1.99)	2.42	(2.69)	2.98	(2.62)	0.0002^b^

*Notes:* GDS = geriatric depression scale. ^a^Fisher’s exact test was performed. ^b^ANOVA test was performed.

### Anthropometric measures and clinical indices of the study participants

Anthropometric measures, such as body mass index (BMI), height-adjusted SMI, weight-adjusted SMI, and clinical indices, including total cholesterol, blood pressure, high-density lipoprotein cholesterol (HDL-C), and fasting blood glucose, are shown in Table 2. In the elderly men and women, the mean height-adjusted SMI in the group having sleep problems and taking sleeping pills were lower than those in the two other groups (*P* = 0.0334 and 0.0072, respectively). However, the mean BMIs did not significantly differ among the three groups of elderly men and women. The clinical indices of the elderly men and women did not also significantly vary, but the HDL-C in the elderly women differed among the groups with various sleep problems.

**Table 2:** Anthropometric Measures and Clinical Indices of the Study Participants According to the Status of Having Sleep Problems and Taking Sleeping Pills.

	Sleep problems among men						*P* value	Sleep problems among women						
	No (n = 276)		Yes (n = 165)					No (n = 175)		Yes (n = 210)				
	Mean	(SD)	Sleeping pills					Mean	(SD)	Sleeping pills				
			No (n = 83)		Yes (n = 82)					No (n = 108)		Yes (n = 102)		
			Mean	(SD)	Mean	(SD)				Mean	(SD)	Mean	(SD)	*P* value
**Anthropometric measures**														
Weight (kg)	65.2	(9.4)	65.2	(11.1)	62.8	(9.0)	0.1268	56.7	(8.4)	56.5	(10.0)	56.2	(7.4)	0.8939
Height (cm)	163.6	(5.8)	163.6	(5.4)	162.6	(5.3)	0.3292	152.3	(5.8)	151.8	(5.4)	152.9	(5.8)	0.4352
BMI (kg/m^2^)	24.3	(3.1)	24.3	(3.5)	23.7	(3.1)	0.2778	24.5	(3.4)	24.5	(4.0)	24.1	(3.3)	0.6774
Waist circumference (cm)	88.0	(8.5)	87.6	(9.5)	86.4	(8.2)	0.3327	80.8	(8.3)	81.5	(9.0)	80.9	(8.2)	0.8007
Hip circumference (cm)	97.3	(6.3)	96.4	(6.9)	95.4	(5.6)	0.0603	97.0	(6.7)	96.6	(8.1)	95.8	(7.0)	0.4256
height-adjusted SMI (kg/m^2^)	7.57	(0.85)	7.50	(0.86)	7.29	(0.86)	0.0334	6.19	(0.74)	6.25	(0.84)	5.95	(0.61)	0.0072
weight-adjusted SMI (%)	31.3	(3.2)	31.2	(3.4)	31.0	(4.1)	0.7512	25.6	(3.1)	26.0	(4.1)	24.9	(2.8)	0.0780
**Clinical indices**														
Systolic blood pressure (mmHg)	139.1	(16.0)	139.0	(15.6)	135.7	(16.4)	0.2224	136.3	(17.6)	140.3	(18.3)	134.7	(16.7)	0.0535
Diastolic blood pressure (mmHg)	79.4	(11.0)	80.3	(11.3)	76.9	(9.6)	0.0981	76.1	(10.0)	77.7	(10.5)	75.2	(11.0)	0.2015
Total cholesterol (mg/dl)	188.0	(34.8)	181.9	(39.0)	179.9	(31.1)	0.1169	198.5	(33.0)	196.4	(35.0)	197.6	(36.0)	0.8858
Triglyceride (mg/dl)	110.7	(63.5)	112.5	(62.1)	116.7	(80.4)	0.7692	116.1	(61.5)	123.7	(62.0)	129.6	(74.1)	0.2399
HDL-C (mg/dl)	43.8	(14.4)	41.0	(12.7)	42.8	(15.0)	0.2857	51.7	(14.4)	48.3	(13.2)	47.6	(11.6)	0.0253
LDL-C (mg/dl)	114.0	(30.2)	108.8	(31.1)	106.6	(25.9)	0.0852	116.1	(30.4)	115.4	(28.6)	116.2	(31.7)	0.9791
Fasting blood glucose (mg/dl)	109.7	(30.3)	107.6	(23.4)	105.9	(20.5)	0.4981	109.7	(30.3)	107.6	(23.4)	105.9	(20.5)	0.4981
Insulin (uIU/ml)^a^	5.34	(1.74)	5.80	(1.79)	5.79	(1.85)	0.3496	6.26	(1.75)	6.50	(1.84)	6.61	(1.75)	0.7352

*Notes:* BMI = body mass index; SMI = skeletal muscle index; HDL-C = high-density lipoprotein cholesterol; LDL-C = low-density lipoprotein cholesterol. ^a^Natural logarithmic transformation was performed and data were shown as geometric mean.

### Association between height-adjusted SMI and sleep problem status

**Figure 1:**
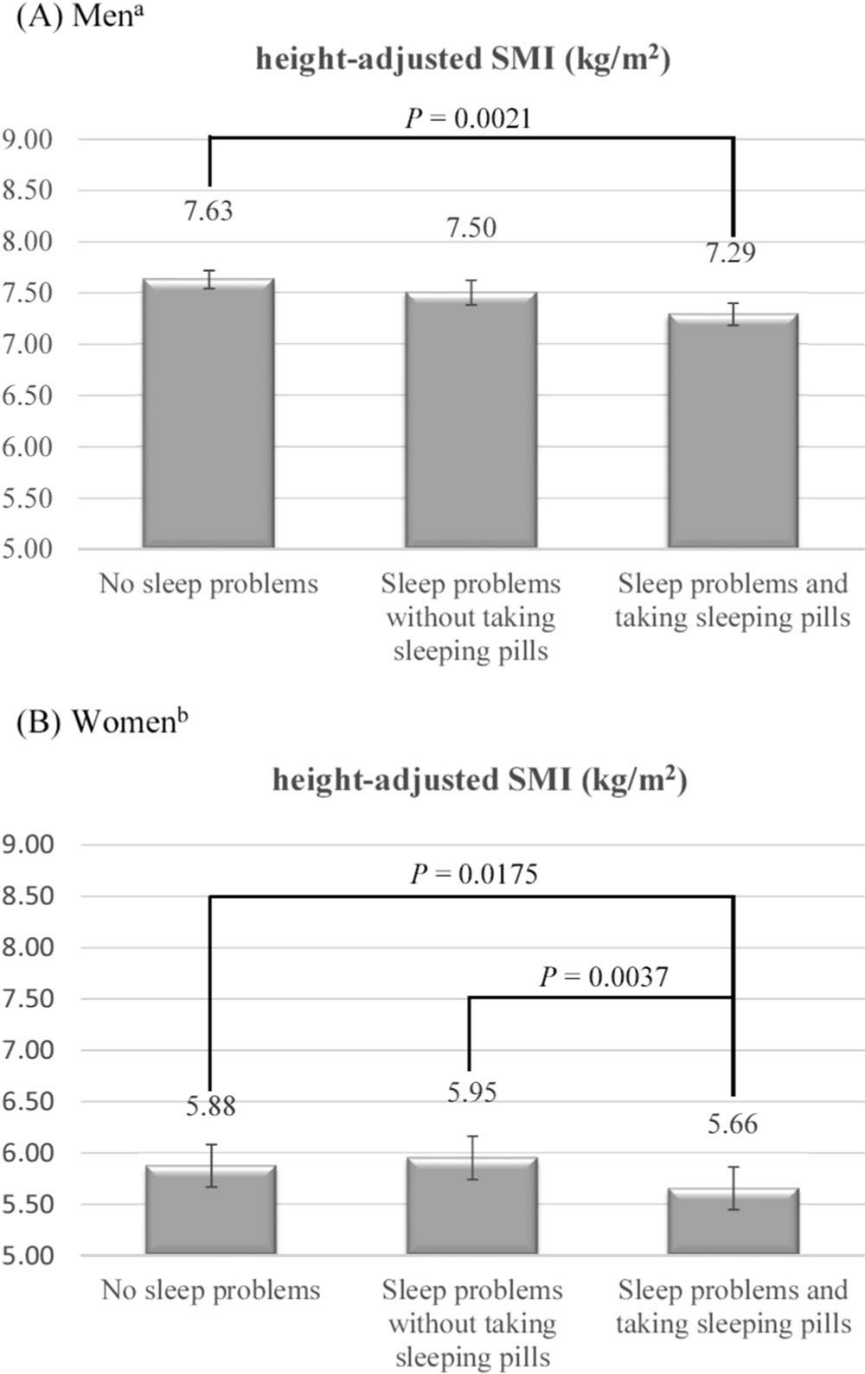
Adjusted means for height-adjusted skeletal muscle mass index (SMI) among (**A**) men and (**B**) women according to the status of having sleep problems and taking sleeping pills. ^a^Adjusted for age, diabetes mellitus, hyperlipidemia, and stroke among men. ^b^Adjusted for age, alcohol consumption, and GDS score among women. These possible confounders were identified by the directed acyclic graphs (DAGs).

We used multivariate linear regression to separately assess the independent relationship between height-adjusted SMI and sleep problem status in elderly men and women (Fig. 1). After adjustments for age, diabetes mellitus, hyperlipidemia, and stroke, which were identified by the directed acyclic graph (DAG)^17^, the adjusted mean height-adjusted SMI of the elderly men having sleep problems and taking sleeping pills was significantly lower than that of the elderly men without sleep problems (7.29 vs. 7.63 kg/m^2^). After adjustments for age, alcohol consumption, and GDS score, which were identified by the DAG, the adjusted mean height-adjusted SMI of the elderly women having sleep problems and taking sleeping pills was 5.66 kg/m^2^, which was significantly lower than that of the elderly women who were having sleep problems but were not taking sleeping pills (5.95 kg/m^2^) and was also significantly lower than that of the elderly women who were not experiencing sleep problems (5.88 kg/m^2^).

### Combined effects of having sleep problems and taking sleeping pills

We further evaluated the combined effects of having sleep problems and taking sleeping pills on physical performance, such as walking speed, grip strength, time up and go, leg press, chair stand, and squat (Table 3). After adjustments for possible confounders, the adjusted means of walking speed (*P* = 0.0392), grip strength (*P* = 0.0530), and number of squats (*P* = 0.0166) for the elderly men having sleep problems and taking sleeping pills were lower than those without sleep problems. After adjustments for possible confounders of the elderly women were obtained, no differences in the adjusted means of these physical performance indicators were observed in the groups with various sleep problems.

**Table 3:** Physical performances in study subjects by sleep problem and taking sleeping pills status.

Gender	Sleep problems	Taking sleeping pills	Walking speed (m/s)	Grip strength (kg)	Time up and go (s)	Leg press / weight (%)	Chair test three times (s)	Squat test (times/20 s)
			β (SE)^a,b^	β (SE)^a,b^	β (SE)^a,b^	β (SE)^a,b^	β (SE)^a,b^	β (SE)^a,b^
Men	−	−	Reference	Reference	Reference	Reference	Reference	Reference
Men	+	−	0.01 (0.03)	−0.14 (0.77)	0.15 (0.57)	−0.07 (0.05)	−0.29 (0.35)	−0.85 (0.58)
Men	+	+	−0.05 (0.03)*	−1.57 (0.81)^c^	0.22 (0.59)	−0.08 (0.05)	0.08 (0.37)	−1.45 (0.60)*
Women	−	−	Reference	Reference	Reference	Reference	Reference	Reference
Women	+	−	−0.02 (0.02)	0.39 (0.58)	0.87 (0.68)	0.01 (0.12)	8.46 (6.19)	−0.62 (0.67)
Women	+	+	−0.02 (0.03)	0.31 (0.60)	−0.16 (0.70)	0.17 (0.12)	−1.62 (6.38)	0.14 (0.69)

^a^Adjusted for age, diabetes mellitus, hyperlipidemia, and stroke among men. ^b^Adjusted for age, alcohol consumption, and GDS score among women. These possible confounders were identified by the directed acyclic graphs (DAGs). ^c^*P* = 0.0530; ^*^P < 0.05.

## Discussion

To our knowledge, the present study is the first to report the combined effects of having sleep problems and taking sleeping pills on the skeletal muscle mass in a sample of Taiwanese community-dwelling elders. This study also revealed that having sleep problems and taking sleeping pills among elderly men were associated with physical performance, such as walking speed, hand grip strength, and number of squats. Consistent with our findings regarding the effects of having sleep problems on lean muscle mass and physical performance, a previous study in Germany found that men with poor sleep quality or sleep efficiency have a significantly high risk of low muscle mass^18^. In Hong Kong, subjective insomnia is associated with slow walking speed and weak hand grip strength among elderly men^19^. Poor upper and lower limb strength were associated with insomnia. Thus, the combined effect of having sleep problems and taking sleeping pills on walking speed and grip strength among elderly men maybe due to poor upper and lower limb strength. However, these previous studies did not consider the combined effects of sleep problems and sleep-inducing drugs.

Some previous findings regarding the effects of sleep-inducing drugs on muscle mass and physical performance were consistent with our results. In Japan, the most common symptoms of taking non-benzodiazepine drugs are impaired balance and falls^20^. In the US, the use of zolpidem for hospitalized patients is independently related to the increased risk of falling^21^. In Finland, elderly adults stopped their long-term use of benzodiazepines, and their handgrip strength and balance are consequently improved^22^. Therefore, the effects of sleep-inducing drugs on physical performance may be due to long-term use of sleeping pills. However, using other types of sleep-inducing drugs, such as tricyclic antidepressants, and melatonin receptor agonist, in older adults were no associated with recurrent falls^23^ and middle-of-the-night balance^24^. Thus, the detailed mechanisms of sleep-inducing drugs on physical performance among elderly should be further investigated.

This study showed that the elder men who having sleep problems and taking sleeping pills has significantly fewer times of squats than those without sleep problems, indicating having sleep problems and taking sleeping pills maybe associated with the balance function and lower limb strength. Previous study found that squat exercise was associated with static and dynamic balance^25^. Furthermore, another study showed that older adults who have sleep problems had significantly higher risk of balance difficulty and falls, even after adjusting for medication use^6^. Therefore, balance function may play an important role among the association between the times of squats and combined effect of having sleep problems and taking sleeping pills.

This study indicated that the combined effect of having sleep problems and taking sleeping pills among elderly men was associated with low skeletal muscle mass, walking speed, and hand grip strength. These three indicators of physical performance have been used to diagnose sarcopenia in the Asian Working Group for Sarcopenia^26^, the European Working Group on Sarcopenia in Older People^27^, and the Foundation for the National Institutes of Health Sarcopenia Project^28^. Therefore, this study finding imply that the combination of having sleep problems and taking sleeping pills among the elderly may be associated with having sarcopenia, which has been supported by a previous study findings demonstrating that elderly adults with sleep duration less than 6 h have a significantly high likelihood of sarcopenia^29^.

The combined effect of having sleep problems and taking sleeping pills on the muscle mass and physical performance of the elderly may be attributed to the change in testosterone levels. Previous studies provided evidence regarding the association of testosterone levels with sleep duration, muscle mass, and physical performance. It has been reported total testosterone and bioavailable testosterone levels are related to sleep duration but are independent of age^14^. Furthermore, muscle mass is linked to total, free, and bioavailable testosterone levels among older men^15,30^ and to free testosterone levels among postmenopausal women^31^. Muscle strength and physical performance are correlated with the total, free, and bioavailable testosterone levels among older men^15,16,32^, but no correlation among older women^33^. Notably, plasma total and free testosterone levels are associated with instrumental activities of daily living (IADL) among elderly men, but no association between testosterone levels and IADL among elderly women is observed^34^. Therefore, the pathophysiological mechanism of this study finding may be explained by the testosterone levels.

Strength of this study is that used two simple self-reported questions for having sleep problems and taking sleeping pills to explore their combined effects on muscle mass and physical performance among elderly. These parameters could be easily applied to sarcopenia screening programs in communities. Some limitations should also be noted in this study. First, the major limitation relevant to the interpretation of our study’s findings is the use of cross-sectional data; thus, we cannot make causal inference about the observed relationships. Second, this study used two self-reported questions to evaluate the sleeping status among the elderly. Sleep duration and types and doses of sleep-inducing drugs were disregarded in this study. Although the measurement was simple, it could determine its significant association with a decrease in lean muscle mass and physical performance. Third, a potential selection bias might exist because only 826 of the 1347 participants were analyzed. We did evaluate this potential bias and found the differences in the joint distributions of age and gender between this study sample of TCHS-E participants and the elderly populations of Taiwan in 2009 were small, ranging from 0.3% to 5.3%. Therefore, this selection bias might be minimal. Fourth, the participants in this study were from a metropolitan city. Thus, our findings should not be generalized to include elders living in rural areas because of the differences in their patterns of sleep problems and physical activities.

In conclusion, the combination of having sleep problems and taking sleeping pills was correlated with the low skeletal muscle mass and physical performance, such as walking speed, hand grip strength, and number of squats, in community-dwelling elders. Our study findings suggested that having sleep problems and taking sleeping pills among the elderly should be useful for health professionals aiming at screening elders at high risks of low muscle mass and physical performance.

## Methods

### Study population and sampling method

The participants in this study were community-dwelling elderly enrolled in the Taichung Community Health Study-Elderly (TCHS-E). The study population included all residents aged ≥65 years in eight administrative neighborhoods in North District, Taichung City, Taiwan, who were registered in June 2009. A total of 3997 older residents in these administrative neighborhoods were invited to participate in this study. We excluded 1247 subjects because of errors on their registry, having moved out of the area, institutionalization, and death. A total of 1347 elders agreed to participate, and their response rate was 49.0%. However, 521 participants were unable to complete the information about sleep problems, skeletal muscle mass, or physical activity. Therefore, 826 subjects were included in this data analysis. The Human Research Committee at the China Medical University Hospital approved this study, all methods were performed in accordance with the relevant guidelines and regulations, and informed consent was obtained from each participant.

### Assessment of appendicular skeletal muscle mass

The mass of the appendicular skeletal muscle was assessed through dual-energy X-ray absorptiometry (GE Lunar DPX Pro, Lunar Corp., Madison, WI, USA), and the appendicular skeletal muscle mass index (SMI) was calculated by dividing the limb muscle mass (kg) by the weight (kg) or square of height (m).

### Assessment of having sleep problems and taking sleeping pills

Having sleep problems and taking sleeping pills were assessed by the self-reported questionnaire. Sleep problem variables comprised four items, including experiencing difficulty in falling asleep, having many dreams, becoming easily awakened, and suffering from sleepiness.

### Measurement of grip strength and physical performance

Hand grip strength was measured with a dynamometer (TTM Dynamometer, Tsutsumi, Tokyo, Japan). Three trials for each hand were carried out. If the difference was more than 3 kg between any two measures in the same hand, then the measurement was repeated again after a rest. The maximum result was used for data analysis. Physical performance tests included a 5-meter walk test, a timed up and go test, a leg press test, a chair stand test, and a squat test. The participants underwent all physical performance tests under the instructions of physical therapists. For the 5-meter walking test, the participants were asked to walk for 5 m as fast as they could, and the time in second to complete the test was recorded. The walking speed (m/sec) was calculated by dividing the distance (5 m) by the recorded time (sec). For the timed up and go test, the participants were instructed to stand up from a sitting position, walk 3 m from that position, turn around, walk back 3 m to the chair, and sit down as fast as possible. The time in second to complete the test was also recorded. For the leg press test, the participants were asked to lift the maximum weight by using both legs 15 times with a successful repetition by a leg press machine (AURA G3-S70, Matrix Fitness System, USA). Then we estimated one-repetition maximum leg press strength by the Brzycki formula. The leg muscle strength was assessed by dividing the maximum lift weight by the body weight, and multiplying by 100%. For the chair stand test, the participants were instructed to fold their arms across their chest and to sit firmly in a chair, then to rise from the chair and sit down. The time in second to complete the test in three repetitions were recorded. For the squat test, the participants were asked to repeatedly squat down for 20 seconds, and the number of squats was recorded.

### Sociodemographic factors, life style behaviors, and mental factors

Sociodemographic characteristics, including age, gender, educational attainment, cigarette smoking, alcohol drinking, recreational physical activity, physician-diagnosed diseases, and fall history, were collected by using questionnaires. For the recreational physical activity, the elders who exercised for at least 30 min three times per week in the last 3 months were classified as having regular exercise. Smoking status was categorized as never, current, and former. Former smokers included those who had smoked at least 100 cigarettes during their lifetime but no longer smoke cigarettes. Mental factors were assessed using the 15-item Geriatric Depression Scale (GDS-15). High scores corresponded to a high level of depression.

### Statistical analysis

Analyses were stratified by gender. The subjects having sleep problems were further classified into two groups according to the status of taking sleeping pills. Categorical variables such as gender, education attainment level, and chronic problems were reported as percentages, whereas continuous variables such as anthropometric measures and clinical indices were presented as mean ± standard deviations (SD). Differences in proportions and means were assessed by using a Chi-square test or an analysis of variance (ANOVA) when appropriate. We used multiple linear regression models to analyze the combined effects of having sleep problems and taking sleeping pills on the skeletal muscle mass and performance after adjustments for possible confounders were made. These possible confounders were identified by the DAGs^17^, which is a 6-step algorithm for determining a proposed set of covariates. These connected variables identified from DAGs were as the potential confounders (Supplementary Fig. S1), including age, diabetes mellitus, hyperlipidemia, and stroke in the regression models of elderly men; and including age, alcohol consumption, and GDS score in the regression models of elderly women. All *P*-values were two sided, and significance level was set at *P* < 0.05. All analyses were performed in SAS version 9.4 (SAS Institute Inc., Cary, NC).

## Supplementary information

Supplementary Figure S1 & Table S1

## Data Availability

The data that support the findings of this study are available from the TCHS-E but restrictions apply to the availability of these data, which were used under license for the current study, and so are not publicly available. Data are however available from the authors upon reasonable request and with permission of the TCHS-E.

## References

[1] Tsou M-T (2013). Prevalence and risk factors for insomnia in community-dwelling elderly in northern Taiwan. Journal of Clinical Gerontology and Geriatrics.

[2] Foley DJ (1995). Sleep complaints among elderly persons: an epidemiologic study of three communities. Sleep.

[3] Chiu HF (1999). Sleep problems in Chinese elderly in Hong Kong. Sleep.

[4] Gray SL (2015). Cumulative use of strong anticholinergics and incident dementia: a prospective cohort study. JAMA internal medicine.

[5] Stone KL (2014). Sleep disturbances and risk of falls in older community-dwelling men: the outcomes of Sleep Disorders in Older Men (MrOS Sleep) Study. Journal of the American Geriatrics Society.

[6] Brassington GS, King AC, Bliwise DL (2000). Sleep problems as a risk factor for falls in a sample of community-dwelling adults aged 64-99 years. Journal of the American Geriatrics Society.

[7] Schubert CR (2002). Prevalence of sleep problems and quality of life in an older population. Sleep.

[8] Pollak CP, Perlick D, Linsner JP, Wenston J, Hsieh F (1990). Sleep problems in the community elderly as predictors of death and nursing home placement. Journal of community health.

[9] Sagayadevan V (2017). Prevalence and correlates of sleep problems among elderly Singaporeans. Psychogeriatrics: the official journal of the Japanese Psychogeriatric Society.

[10] Spira AP, Chen-Edinboro LP, Wu MN, Yaffe K (2014). Impact of sleep on the risk of cognitive decline and dementia. Current opinion in psychiatry.

[11] Jelicic M (2002). Subjective sleep problems in later life as predictors of cognitive decline. Report from the Maastricht Ageing Study (MAAS). International journal of geriatric psychiatry.

[12] Rubenstein LZ, Josephson KR (2002). The epidemiology of falls and syncope. Clinics in geriatric medicine.

[13] Rubenstein LZ (2006). Falls in older people: epidemiology, risk factors and strategies for prevention. Age and ageing.

[14] Goh VH, Tong TY (2010). Sleep, sex steroid hormones, sexual activities, and aging in Asian men. Journal of andrology.

[15] Baumgartner RN, Waters DL, Gallagher D, Morley JE, Garry PJ (1999). Predictors of skeletal muscle mass in elderly men and women. Mechanisms of ageing and development.

[16] Auyeung TW (2011). Testosterone but not estradiol level is positively related to muscle strength and physical performance independent of muscle mass: a cross-sectional study in 1489 older men. European journal of endocrinology.

[17] Shrier I, Platt RW (2008). Reducing bias through directed acyclic graphs. BMC medical research methodology.

[18] Buchmann N (2016). Sleep, Muscle Mass and Muscle Function in Older People. Deutsches Arzteblatt international.

[19] Auyeung TW (2015). Sleep Duration and Disturbances Were Associated With Testosterone Level, Muscle Mass, and Muscle Strength–A Cross-Sectional Study in 1274 Older Men. Journal of the American Medical Directors Association.

[20] Kajiwara A (2016). Safety analysis of zolpidem in elderly subjects 80 years of age or older: adverse event monitoring in Japanese subjects. Aging & mental health.

[21] Kolla BP, Lovely JK, Mansukhani MP, Morgenthaler TI (2013). Zolpidem is independently associated with increased risk of inpatient falls. Journal of hospital medicine.

[22] Nurminen J (2014). Handgrip strength and balance in older adults following withdrawal from long-term use of temazepam, zopiclone or zolpidem as hypnotics. BMC geriatrics.

[23] Marcum ZA (2016). Antidepressant Use and Recurrent Falls in Community-Dwelling Older Adults: Findings From the Health ABC Study. The Annals of pharmacotherapy.

[24] Zammit G, Wang-Weigand S, Rosenthal M, Peng X (2009). Effect of ramelteon on middle-of-the-night balance in older adults with chronic insomnia. Journal of clinical sleep medicine: JCSM: official publication of the American Academy of Sleep Medicine.

[25] Simao AP (2012). Functional performance and inflammatory cytokines after squat exercises and whole-body vibration in elderly individuals with knee osteoarthritis. Archives of physical medicine and rehabilitation.

[26] Chen LK (2014). Sarcopenia in Asia: consensus report of the Asian Working Group for Sarcopenia. Journal of the American Medical Directors Association.

[27] Cruz-Jentoft AJ (2010). Sarcopenia: European consensus on definition and diagnosis: Report of the European Working Group on Sarcopenia in Older People. Age and ageing.

[28] Studenski SA (2014). The FNIH sarcopenia project: rationale, study description, conference recommendations, and final estimates. The journals of gerontology. Series A, Biological sciences and medical sciences.

[29] Chien MY, Wang LY, Chen HC (2015). The Relationship of Sleep Duration with Obesity and Sarcopenia in Community-Dwelling Older Adults. Gerontology.

[30] Iannuzzi-Sucich M, Prestwood KM, Kenny AM (2002). Prevalence of sarcopenia and predictors of skeletal muscle mass in healthy, older men and women. The journals of gerontology. Series A, Biological sciences and medical sciences.

[31] Gower BA, Nyman L (2000). Associations among oral estrogen use, free testosterone concentration, and lean body mass among postmenopausal women. The Journal of clinical endocrinology and metabolism.

[32] Schaap LA (2005). The association of sex hormone levels with poor mobility, low muscle strength and incidence of falls among older men and women. Clinical endocrinology.

[33] Carcaillon L (2012). Sex differences in the association between serum levels of testosterone and frailty in an elderly population: the Toledo Study for Healthy Aging. PloS one.

[34] Fukai S (2009). Association of plasma sex hormone levels with functional decline in elderly men and women. Geriatrics & gerontology international.

