# Supplementary material for: Combined Effects of Having Sleep Problems and Taking Sleeping Pills on the Skeletal Muscle Mass and Performance of Community-Dwelling Elders
Source: Sci Rep. 2019 Sep 24;9:13760. doi: 10.1038/s41598-019-50295-w (PMC6760224; doi:10.1038/s41598-019-50295-w)
Supplement: Supplementary file 1 — Supplementary Figure S1 & Table S1 [file 41598_2019_50295_MOESM1_ESM.pdf]

Combined Effects of Having Sleep Problems and Taking Sleeping Pills on the Skeletal Muscle Mass and Performance of Community-Dwelling Elders

Chuan-Wei Yang[1], Chia-Ing Li[1,2], Tsai-Chung Li[3,4], Chiu-Shong Liu[2,5], Chih-Hsueh Lin[2,5], Wen-Yuan Lin[2,5], Cheng-Chieh Lin[2,4,5]

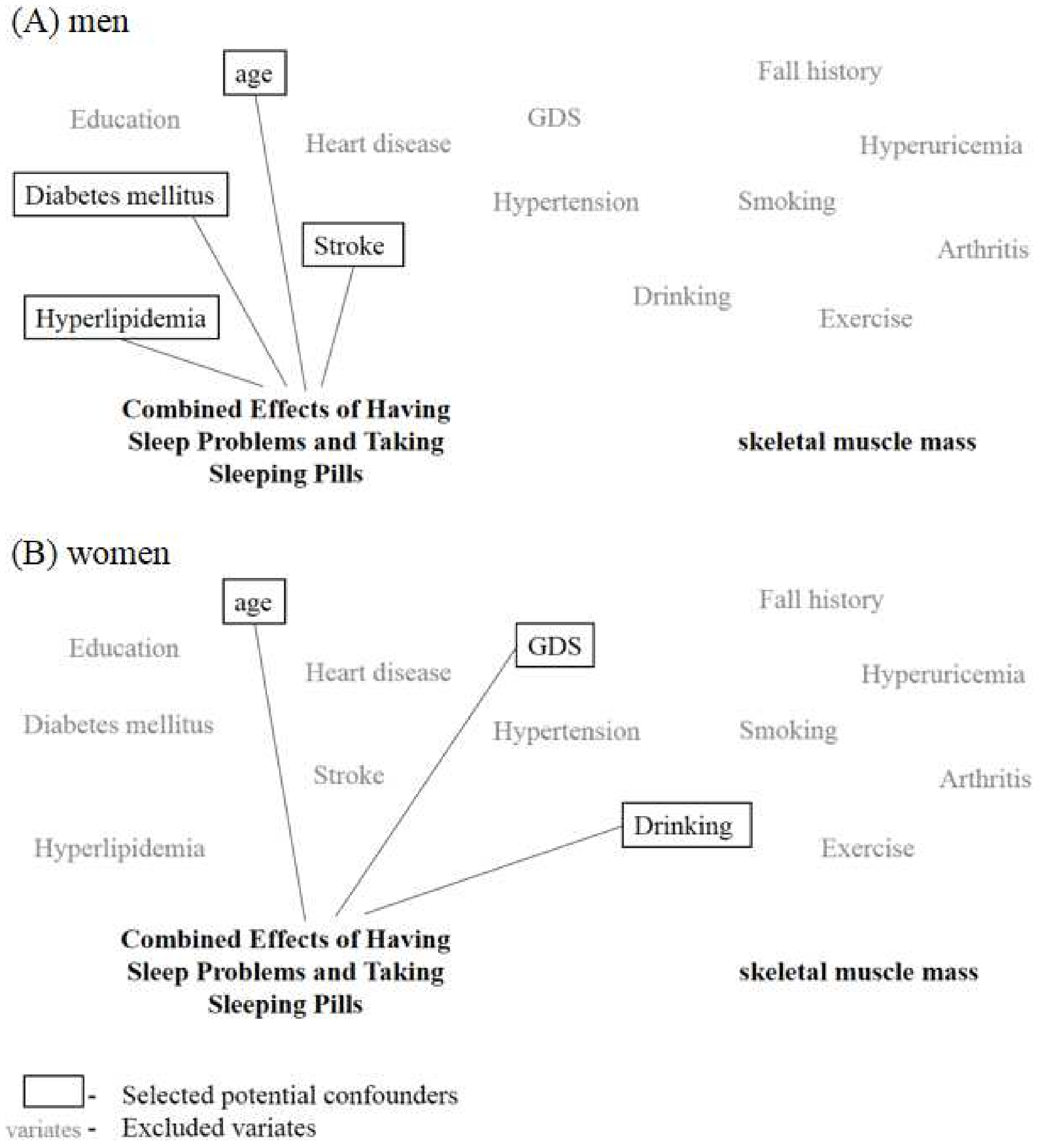

Supplementary Figure S1. Select potential confounders by DAGs among (A) men and

(B) women. First, we listed all variables which should not be descendants of the combined effect of having sleep problems and taking sleeping pills, and next deleted variables which are non-ancestors of the combined effect of having sleep problems and taking sleeping pills, or skeletal muscle mass. Then, we deleted the line emanating from the combined effect of having sleep problems and taking sleeping pills, and connected any two variables which connected to the same variables. Finally, we stripped all arrowheads from lines, and deleted all lines between the covariates.

Supplementary Table S1. The correlation table of all critical variables.

Men / Women	Sleep Problems	Sleeping pills	Hypertension	Diabetes	Hyperlipidemia	Stroke	GDS	Smoking habits	Drinking habits	Exercise habits	FALL	age	SMI	Walking speed (m/s)	Grip strength (kg)	Time up and go (s)	Leg press / weight (%)	Chair test three times (s)	Squat test (times/20s)
**Sleep Problems**	1.0000	0.5186	-0.0113	-0.0171	0.0552	0.0566	0.0825	0.0284	0.0566	-0.0066	0.0365	-0.1158	-0.0953	0.0060	-0.0205	0.0104	-0.0397	-0.0389	-0.0457
		<.0001	0.8585	0.7877	0.3832	0.3710	0.1916	0.6540	0.3711	0.9171	0.5637	0.0665	0.1314	0.9250	0.7462	0.8693	0.5304	0.5393	0.4705
**Sleeping pills**	0.4702	1.0000	0.1611	0.1604	0.1143	0.1769	-0.0282	0.0863	0.1273	-0.0427	-0.0442	0.0179	-0.0902	-0.1580	-0.1375	0.1353	-0.0851	0.0310	-0.0920
	<.0001		0.0104	0.0108	0.0701	0.0049	0.6556	0.1719	0.0436	0.4999	0.4852	0.7779	0.1532	0.0120	0.0291	0.0318	0.1779	0.6241	0.1454
**Hypertension**	-0.0854	-0.0721	1.0000	0.8081	0.2656	0.2494	-0.0326	0.0231	0.0187	-0.0755	0.1830	0.1181	0.0198	-0.2023	-0.1482	0.1696	-0.2216	0.1335	-0.2069
	0.2091	0.2891		<.0001	<.0001	<.0001	0.6068	0.7148	0.7672	0.2326	0.0036	0.0613	0.7548	0.0012	0.0186	0.0070	0.0004	0.0341	0.0010
**Diabetes**	-0.0321	-0.0569	0.8248	1.0000	0.2074	0.2119	-0.0403	0.0440	-0.0353	-0.0795	0.1621	0.1113	0.0506	-0.2552	-0.1774	0.2319	-0.2506	0.1218	-0.2165
	0.6375	0.4035	<.0001		0.0009	0.0007	0.5242	0.4870	0.5771	0.2085	0.0099	0.0778	0.4235	<.0001	0.0047	0.0002	<.0001	0.0535	0.0005
**Hyperlipidemia**	-0.0116	0.0189	0.1120	0.1137	1.0000	0.0495	-0.0282	-0.0488	-0.0075	0.0085	0.1030	-0.0580	0.1107	0.0030	0.0508	-0.0246	0.0222	0.0025	0.0685
	0.8651	0.7819	0.0990	0.0942		0.4337	0.6556	0.4406	0.9059	0.8927	0.1027	0.3594	0.0794	0.9624	0.4220	0.6981	0.7256	0.9691	0.2791
**Stroke**	0.0193	0.0513	0.2397	0.2978	-0.0286	1.0000	-0.0170	-0.0580	0.0400	-0.1226	0.0599	-0.0668	0.0796	-0.2664	-0.1743	0.2987	-0.1603	0.0782	-0.1660
	0.7774	0.4509	0.0004	<.0001	0.6745		0.7886	0.3589	0.5274	0.0519	0.3437	0.2911	0.2078	<.0001	0.0055	<.0001	0.0108	0.2161	0.0083
**GDS**	0.0891	0.2080	-0.0636	-0.0771	-0.0966	-0.0318	1.0000	0.0885	0.0877	-0.1181	-0.0270	-0.0094	-0.0593	0.0798	-0.0276	-0.0372	-0.0698	0.0048	-0.0263
	0.1898	0.0020	0.3504	0.2573	0.1552	0.6406		0.1614	0.1652	0.0612	0.6696	0.8826	0.3482	0.2069	0.6623	0.5569	0.2699	0.9392	0.6782
**Smoking habits**	0.0891	-0.0107	0.1096	0.0756	-0.0966	-0.0318	0.1812	1.0000	0.2831	-0.1032	0.0660	0.0685	-0.1747	0.0122	0.0019	-0.0356	-0.0943	-0.0149	-0.1114
	0.1898	0.8752	0.1065	0.2661	0.1552	0.6406	0.0073		<.0001	0.1022	0.2964	0.2788	0.0054	0.8477	0.9755	0.5734	0.1355	0.8144	0.0775
**Drinking habits**	-0.1171	-0.0984	-0.1087	-0.1317	-0.1652	-0.0544	0.0849	0.2099	1.0000	-0.0380	0.0854	-0.0470	0.1027	-0.0187	0.0753	0.0004	0.0195	-0.0085	0.0031
	0.0845	0.1476	0.1096	0.0521	0.0146	0.4245	0.2120	0.0018		0.5479	0.1766	0.4579	0.1040	0.7681	0.2335	0.9946	0.7585	0.8928	0.9607
**Exercise habits**	-0.1110	-0.0199	0.0370	0.0791	0.0959	-0.1644	0.0091	-0.0643	0.0514	1.0000	0.0176	-0.0302	0.0989	0.1827	0.0757	-0.1806	0.1390	-0.0861	0.0882
	0.1021	0.7701	0.5865	0.2447	0.1584	0.0151	0.8938	0.3446	0.4502		0.7811	0.6331	0.1174	0.0036	0.2309	0.0040	0.0274	0.1730	0.1627
**FALL**	-0.0012	0.0245	0.0591	0.0939	-0.0532	0.1788	0.1093	-0.0272	0.0868	-0.0560	1.0000	0.0386	-0.1786	-0.2382	-0.1103	0.1351	-0.2627	0.1344	-0.2199
	0.9857	0.7188	0.3854	0.1671	0.4345	0.0081	0.1076	0.6892	0.2017	0.4104		0.5423	0.0045	0.0001	0.0804	0.0321	<.0001	0.0330	0.0004
**age**	0.1152	0.0170	0.1471	0.1943	-0.0132	0.1018	0.0939	-0.0800	-0.0525	-0.1036	0.1070	1.0000	-0.2691	-0.3402	-0.3977	0.2479	-0.3452	0.1196	-0.2534
	0.0896	0.8026	0.0299	0.0040	0.8461	0.1339	0.1671	0.2395	0.4407	0.1274	0.1154		<.0001	<.0001	<.0001	<.0001	<.0001	0.0581	<.0001
**SMI**	-0.0527	-0.1393	-0.0211	0.0185	0.1331	-0.0830	-0.1229	-0.0853	-0.0293	0.2086	-0.0658	-0.2451	1.0000	0.1430	0.2897	-0.0670	0.3931	0.0408	0.2085
	0.4386	0.0400	0.7571	0.7860	0.0498	0.2223	0.0702	0.2094	0.6676	0.0020	0.3337	0.0003		0.0232	<.0001	0.2891	<.0001	0.5188	0.0009
**Walking speed (m/s)**	-0.0970	-0.0197	-0.1450	-0.2514	0.0956	-0.3217	-0.1088	-0.1029	-0.0083	0.2638	-0.2101	-0.4858	0.2531	1.0000	0.4479	-0.7624	0.5230	-0.3788	0.6051
	0.1536	0.7720	0.0324	0.0002	0.1597	<.0001	0.1092	0.1298	0.9026	<.0001	0.0018	<.0001	0.0002		<.0001	<.0001	<.0001	<.0001	<.0001
**Grip strength (kg)**	0.0204	0.0444	-0.1890	-0.2247	0.0174	-0.2060	-0.0644	-0.0068	0.0816	0.1818	-0.1239	-0.4037	0.3388	0.4591	1.0000	-0.3333	0.4850	-0.1349	0.3871
	0.7647	0.5148	0.0051	0.0008	0.7989	0.0022	0.3443	0.9209	0.2301	0.0071	0.0678	<.0001	<.0001	<.0001		<.0001	<.0001	0.0323	<.0001
**Time up and go (s)**	0.0836	-0.0705	0.2179	0.2318	-0.1027	0.1295	0.0444	0.0489	-0.0092	-0.2223	0.0671	0.3166	-0.1820	-0.6178	-0.2761	1.0000	-0.4238	0.3077	-0.5274
	0.2192	0.3004	0.0012	0.0006	0.1307	0.0563	0.5143	0.4728	0.8930	0.0010	0.3238	<.0001	0.0071	<.0001	<.0001		<.0001	<.0001	<.0001
**Leg press / weight**	0.0564	0.1205	-0.0739	-0.1063	-0.0344	-0.0839	-0.0426	-0.0346	0.0119	-0.0563	0.0201	-0.1243	0.0143	0.0786	0.1862	-0.0589	1.0000	-0.2450	0.5591
**(%)**	0.4071	0.0758	0.2772	0.1177	0.6132	0.2172	0.5312	0.6114	0.8616	0.4084	0.7680	0.0670	0.8339	0.2476	0.0058	0.3869		<.0001	<.0001
**Chair test three**	0.0713	-0.0406	0.1708	0.1424	-0.0451	-0.0022	-0.0029	-0.0065	-0.0157	-0.1365	-0.0386	0.2703	-0.1497	-0.2259	-0.1589	0.4743	-0.0572	1.0000	-0.3569
**times (s)**	0.2946	0.5510	0.0115	0.0356	0.5073	0.9742	0.9663	0.9235	0.8175	0.0441	0.5713	<.0001	0.0271	0.0008	0.0189	<.0001	0.4009		<.0001
**Squat test**	-0.0490	0.0091	-0.1352	-0.1412	0.0725	-0.1944	-0.1099	-0.0987	0.0417	0.1612	-0.1415	-0.2964	0.2518	0.4954	0.2617	-0.3563	0.0564	-0.1549	1.0000
**(times/20s)**	0.4714	0.8935	0.0461	0.0372	0.2866	0.0040	0.1055	0.1462	0.5402	0.0172	0.0368	<.0001	0.0002	<.0001	<.0001	<.0001	0.4076	0.0221	

The data in the black box is shown by Phi coefficient and the others data is shown by point-biserial correlation coefficient. The data among men is shown on the right up of table and the data among women is shown on the left down of table.
